# Trade-offs among brain structural network characteristics across the cognitive decline process in cerebral small vessel disease

**DOI:** 10.3389/fnagi.2024.1465181

**Published:** 2024-11-28

**Authors:** Yao Wang, Mianxin Liu, Yuewei Chen, Yage Qiu, Xu Han, Qun Xu, Dinggang Shen, Yan Zhou

**Affiliations:** ^1^Department of Radiology, Renji Hospital, School of Medicine, Shanghai Jiao Tong University, Shanghai, China; ^2^Shanghai Artificial Intelligence Laboratory, Shanghai, China; ^3^Department of Neurology, Renji Hospital, School of Medicine, Shanghai Jiao Tong University, Shanghai, China; ^4^School of Biomedical Engineering, ShanghaiTech University, Shanghai, China

**Keywords:** cerebral small vessel disease, cognitive impairment, diffusion tensor imaging, network efficiency, brain reserve

## Abstract

**Objectives:**

To investigate the potential trade-offs among brain structural network characteristics across different stages of cognitive impairment in cerebral small vessel disease (CSVD) based on diffusion tensor imaging (DTI).

**Methods:**

A total of 264 CSVD patients, including 95 patients with non-cognitive impairment (NCI), 142 with mild cognitive impairment (MCI), 27 with vascular dementia (VaD), and 30 healthy controls (HC) underwent cognitive test and brain diffusion magnetic resonance imaging (MRI). The brain structural network was constructed using connections between 90 cortical and subcortical regions. Network characteristics, including sparsity, redundancy, global efficiency (Eg), and local efficiency (Eloc), were calculated.

**Results:**

Sparsity and redundancy significantly declined in the NCI group compared to the HC group. Eg was significantly reduced in the MCI group compared to the NCI group. All network characteristics declined in the VaD group compared to the MCI group. In the NCI group, both sparsity and redundancy were significantly positively correlated with Montreal Cognitive Assessment (MoCA). In the MCI group, there was significant positive correlation between Eg and MoCA. In the VaD group, there was significant negative correlation between Eloc and MoCA. When controlling for sparsity, Eloc exhibited a significant negative correlation with Eg in all three CSVD groups, while redundancy displayed a significant negative correlation with Eg specifically in MCI group.

**Conclusion:**

Our study provides evidence for the heterogeneous alterations in brain structural network across different stages of cognitive impairment in CSVD. The disconnection of brain structural network at NCI stage is mainly the loss of redundant connections. The decline of Eg is the vital factor for cognitive impairment at MCI stage. The decline of all network characteristics is the prominent manifestation at VaD stage. Throughout the cognitive decline process in CSVD, there are trade-offs among the brain network wiring cost, integration, and segregation.

## Introduction

As the major cause of vascular cognitive impairment, cerebral small vessel disease (CSVD) has been progressively acknowledged as a dynamic whole brain disease because of the intricate and widespread microvascular pathology, coupled with variable rates of progression of the related lesions (Hamilton et al., 2021; Jokinen et al., 2020; Shi and Wardlaw, 2016). With the development of neuroimaging research on CSVD, several consistent imaging features, including recent small subcortical infarcts, lacunes, white matter hyperintensities (WMH), perivascular spaces, microbleeds, and brain atrophy have been identified as factors associated with cognitive decline (Wardlaw et al., 2013b). Neuroimaging is highly expected to be used as an alternative to complex neuropsychological scales such as Montreal Cognitive Assessment (MoCA), Mini-Mental State Examination (MMSE), and various cognitive domain-specific scales, to provide a more objective and patient-tolerated assessment method. Nonetheless, to date, there remains a paucity of a unified and efficacious imaging strategy for assessing the cognitive performance of CSVD patients, primarily due to the intricate nature of lesion combinations and their distribution (Jokinen et al., 2020; Wardlaw et al., 2013b). Moreover, diffusion tensor imaging (DTI) studies have suggested that CSVD induces extensive microstructural changes, which cannot be detected by conventional neuroimaging.

The brain network theory provides an additional avenue for a comprehensive assessment and understanding of the progression of cognitive impairment related to CSVD from a global perspective. Recent studies have demonstrated that brain structural network efficiency can serve as a sensitive imaging indicator for evaluating cognitive dysfunction in CSVD (Boot et al., 2020; Tuladhar et al., 2020). The brain structural network efficiency has also been proposed to mediate cognitive deficits along with various macro and microstructural brain injuries, providing a systemic perspective on brain lesions (Du et al., 2019; Lawrence et al., 2014). The global efficiency gauges the network’s capability for global information integration, which has been reported to be associated with cognitive impairment in CSVD (Tuladhar et al., 2016a). Local efficiency assesses network segregation, specialization, and fault tolerance, which has also been linked to cognitive impairment in CSVD (Latora and Marchiori, 2001). However, alterations in network efficiency may not fully account for the impact of CSVD on cognition, as observed in cases with significant CSVD lesions but maintain normal cognitive function. It is possible that brain reserve and compensatory mechanisms play pivotal roles in the pathogenesis of CSVD, suggesting that the effects of CSVD on cognitive impairment are not linearly imposed (Telgte et al., 2018).

Brain reserve has been used to reflect the neurobiological status of the brain (numbers of neurons, synapses, etc.) at any point in time (Stern et al., 2020). The redundancy is defined as “the amount/strength of alternate connections” within a network, which is considered the most likely measure related to the brain reserve (Navlakha et al., 2014; Tononi et al., 1999). Previous studies have suggested redundancy as a neuroprotective mechanism in neurodegenerative diseases such as Parkinson’s disease (PD), Alzheimer’s disease (AD), and normal aging (Arkadir et al., 2014; Langella et al., 2021; Sadiq et al., 2021). Brain redundancy is expected to maintain brain efficiency and cognitive functions. However, in the individual brains, network efficiency (shortcuts in the network) and network redundancy (multiple alternative paths, not guaranteed by shortcuts) should both be implemented under the given wiring costs (the number of connections, measured by network sparsity). Previous studies have indicated that CSVD-related damage to white matter integrity results in a reduction in the sparsity of the white matter network (Reber et al., 2021; Tuladhar et al., 2016a). Therefore, progressively decreasing wiring costs during CSVD progression should force a trade-off between brain network topologies that reflects the processes by which the brain maintains cognitive function despite of existing pathology (Ma et al., 2023). In other words, when the sparsity of brain structural network decreases due to various CSVD-related brain injuries, the efficiency of brain network may not decrease linearly. Because of brain reserve such as redundancy or compensatory mechanisms, the efficiency of brain network may decline at different rates in different stages of the disease, thus exhibiting different cognitive functional states.

So far, the relationship among network sparsity, global and local efficiency, redundancy, and cognitive functions has not been elucidated across different stages of cognitive impairment in CSVD. Analyzing the changes of these brain structural network configurations in different stages of cognitive impairment will help us to understand the robustness of the brain network in CSVD patients.

## Methods

### Participants

A total of 264 patients with CSVD were recruited. CSVD was defined as subcortical WMH on T2-weighted images with at least one lacunar infarct, following the criteria suggested by Galluzzi et al. (2005). Another 30 healthy controls (HC) were recruited from the community. Each participant underwent a standard evaluation, including complete sociodemographic and clinical data collection, such as vascular risk factors, neurological examination, neuropsychological assessment, and magnetic resonance imaging (MRI) examination. The inclusion criteria were as follows: (1) at least 6 years of education; (2) age 50 to 85 years; (3) informed consent form signed by the participant. The following exclusion criteria were applied: (1) cortical and/or cortico-subcortical non-lacunar territorial infarcts and watershed infarcts; (2) neurodegenerative diseases (including PD and AD); (3) signs of normal pressure hydrocephalus; (4) specific causes of WMH (e.g., metabolic, toxic, infectious, multiple sclerosis, brain irradiation); (5) alcoholic encephalopathy or illicit drug use; (6) major depression (Hamilton Depression Rating Scale [HDRS] ≥ 18); (7) severe cognitive impairment (inability to perform the neuropsychological test or undergo the whole MRI scan); (8) MRI safety contraindications and claustrophobia. All patients underwent laboratory examinations to exclude systemic or other neurological diseases mentioned in the above exclusion criteria. Protocols were approved by the Research Ethics Committee. Participants gave their prior written informed consent.

### Cognitive measures

Neuropsychological assessments were performed within 1 week of the MRI examination. No patients experienced any transient ischemic attacks or strokes between the MRI examination and the neuropsychological evaluation. The MoCA and MMSE were used to assess the overall cognitive performance. Moreover, a comprehensive battery of neuropsychological tests was designed to evaluate the four key cognitive domains as described in previous studies (Hachinski et al., 2006; Xu et al., 2014). These tests were as follows: (1) attention and executive function: Trail-Making Tests A and B (TMT-A and TMT-B), Stroop color-word test (Stroop C-T), and verbal fluency test (VFT); (2) visuospatial function: Rey-Osterrieth Complex Figure Test (copy); (3) language function: Boston Naming Test (30 items); (4) memory function: auditory verbal learning test (short and long-delayed free recall). Functional ability was assessed using the Katz basic activities of daily living (BADL) and Lawton and Brody instrumental activities of daily living (IADL) scales. The norms used here were based on the mean scores of each measurement from a sample of typical elderly community members in Shanghai, China (Guo et al., 2007). Cognitive impairment was defined as 1.5 standard deviations below the normative mean on any neuropsychological test. According to the AHA Statement on Vascular Contributions to Cognitive Impairment and Dementia (Gorelick et al., 2011), vascular dementia (VaD) diagnosis was based on a decline in cognitive function from a prior baseline, and a deficit in performance in ≥2 cognitive domains that were of sufficient severity to affect the subject’s ability to perform daily activities, which were independent of the motor/sensory sequelae of the vascular event. The diagnostic criteria of mild cognitive impairment (MCI) included: (1) normal instrumental activity of daily living, (2) does not meet the criteria for dementia, (3) mild quantifiable cognitive decline within one or more cognitive domains (e.g., attention-executive function, memory, language, or visuospatial function). CSVD with non-cognitive impairment (NCI) was defined when CSVD patients scored within the normal range on all neuropsychological tests. A total of 320 CSVD patients were initially collected. Excluding patients who were unable to cooperate to complete neuropsychological scales or neuroimaging studies, and patients with poor image quality by visual exclusion by experienced radiologists, 27 VaD patients, 142 MCI patients, 95 NCI patients, and 30 HC participants were finally included in this study.

### Image acquisition

All MRI data were obtained using a 3.0 T MRI scanner (Signa HDxt; GE HealthCare, Milwaukee, WI, United States) equipped with an eight-channel phased array head coil. The following whole-brain sequences were obtained: (1) Sagittal T1-weighted images covering the whole brain were acquired by the 3D-fast spoiled gradient recalled echo (SPGR) sequence (repetition time [TR] = 5.6 ms, echo time [TE] = 1.8 ms, inversion time [TI] = 450 ms, flip angle = 15^°^, slice thickness = 1.0 mm, number of slices = 156, gap = 0, field of view [FOV] = 256 mm × 256 mm, and matrix = 256 × 256, scanning time = 3′53″); (2) T2-fluid attenuated inversion recovery (FLAIR) sequences (TR = 9,075 ms, TE = 150 ms, TI = 2,250 ms, FOV = 256 mm × 256 mm, matrix = 256 × 256, slice thickness = 2 mm, and number of slices = 66, scanning time = 7′18″); (3) DTI (TR = 17,000 ms, TE = 89.8 ms, slice thickness = 2 mm, gap = 0, FOV = 256 mm × 256 mm, number of slices = 66, matrix = 128 × 128, and 20 diffusion-weighted directions with b value = 1,000 s/mm^2^, scanning time = 6′14″). The total scanning time was 17′27″.

### Brain structural network analysis

The diffusion MRI dataset was analyzed using a pipeline toolbox, PANDA v1.3.1,^1^ which is based on FSL tools (Wang et al., 2017). In the pipeline, skull-stripping with the brain extraction tool (BET) was done to extract the brain tissue for the b0 image in each participant. Eddy current-induced distortions and head motion artifacts were corrected by registering each raw diffusion-weighted image to the b0 image with an affine transformation (Jenkinson and Smith, 2001; Neeman et al., 1991).

Diffusion tensor tractography was applied to build the brain structural network for each individual. Whole brain WM tracts were subsequently reconstructed using the tracking (FACT) algorithm with an FA threshold of 0.2 and a tracking turning angular threshold of 45° (Tay et al., 2019). The whole brain was parcellated into 90 regions of interest according to the automated anatomical labeling (AAL) template. Each region was considered to be one node of the network. The weighted connectivity of the network was defined by using the fiber number between two connected nodes.

Sparsity is defined as the ratio of existing connectivity (with non-zero weights) to the number of connectivity in a fully connected network. The efficiency of a pair of nodes in a graph is the multiplicative inverse of the shortest path distance between the nodes. The local efficiency of a node in the graph is the average global efficiency of the subgraph induced by the neighbors of the node. The average local efficiency (Eloc) is the average of the local efficiencies of each node. The average global efficiency (Eg) of a graph is the average efficiency of all pairs of nodes. Network redundancy is defined as “the number of alternative connections” within a network. The number of independent paths (paths that do not involve the shared nodes) between each node and the other nodes was calculated, and the redundancy of the whole brain network was obtained after adding up the number.

### Statistical analysis

Data analyses and statistics were performed using GraphPad Prism version 8.0 and SPSS version 26. Normally distribution data were compared using the t-test, and non-normally distributed data were analyzed using the Wilcoxon rank-sum test. The Chi-square test was used to compare the gender. One-way analysis of covariance (ANCOVA) was used to analyze the differences of demographic and network characteristics among the HC, NCI, MCI, and VaD groups. Because of the unbalanced group sizes, ANCOVA was computed with type III sums of squares. In case of a significant result, between-group *post-hoc* comparisons were conducted with the Tukey test for multiple comparisons correction. Linear regression analysis was conducted within each CSVD group to determine whether the network characteristics were associated with cognitive function after controlling for demographic. The correlation between the network characteristics was analyzed using partial correlation analysis within each CSVD group. A two-tailed *p*-value of <0.05 was considered statistically significant.

## Results

### Demographic and network characteristics, and cognitive function among the different groups

The demographic and brain network characteristics and cognitive function of the participants are presented in Table 1. There were significant differences in age and education among the groups (*F* = 13.62, *p* < 0.0001; *F* = 9.114, *p* < 0.0001), but no significant differences in gender (χ^2^ = 5.890, *p* = 0.1171). There were significant differences in MoCA, MMSE, network sparsity, redundancy, Eloc and Eg among the groups (*F* = 88.42; *F* = 88.32; *F* = 27.78; *F* = 26.14; *F* = 19.17; *F* = 31.14; all *p*-values <0.0001).

**Table 1:** Statistics for demographic and brain network characteristics, and cognitive function among the groups.

	HC	NCI	MCI	VaD	Test statistic	*p* value
MoCA	26.39 ± 1.85	25.71 ± 2.66	21.65 ± 3.62	14.38 ± 4.13	F = 88.42	**<0.0001**
MMSE	29.00 ± 1.18	28.53 ± 1.43	27.30 ± 2.10	19.38 ± 6.86	F = 88.32	**<0.0001**
Age	65.83 ± 4.97	65.62 ± 7.36	65.32 ± 7.04	74.52 ± 7.53	F = 13.62	**<0.0001**
Education	11.57 ± 2.64	11.48 ± 3.09	9.86 ± 2.70	9.26 ± 3.22	F = 9.114	**<0.0001**
Females, n(%)	9(0.30)	16(0.17)	36(0.25)	10(0.37)	χ^2^ = 5.890	0.1171
Sparsity	0.128 ± 0.017	0.119 ± 0.015	0.114 ± 0.015	0.094 ± 0.008	F = 27.78	**<0.0001**
Redundancy	8.60 ± 1.27	7.97 ± 1.08	7.63 ± 1.16	6.16 ± 0.65	F = 26.14	**<0.0001**
Eloc	0.696 ± 0.024	0.682 ± 0.028	0.677 ± 0.029	0.643 ± 0.027	F = 19.17	**<0.0001**
Eg	0.483 ± 0.022	0.470 ± 0.024	0.460 ± 0.026	0.424 ± 0.022	F = 31.14	**<0.0001**

HC, healthy control; NCI, non-cognitive impairment; MCI, mild cognitive impairment; VaD, vascular dementia; MoCA, Montreal Cognitive Assessment; MMSE, mini-mental state examination; Eloc, local efficiency; Eg, global efficiency. Descriptive statistics are presented as mean ± SD or *n* (%). Bold *p* values were *p* < 0.05.

### Between-group *post-hoc* comparisons

**Figure 1:**
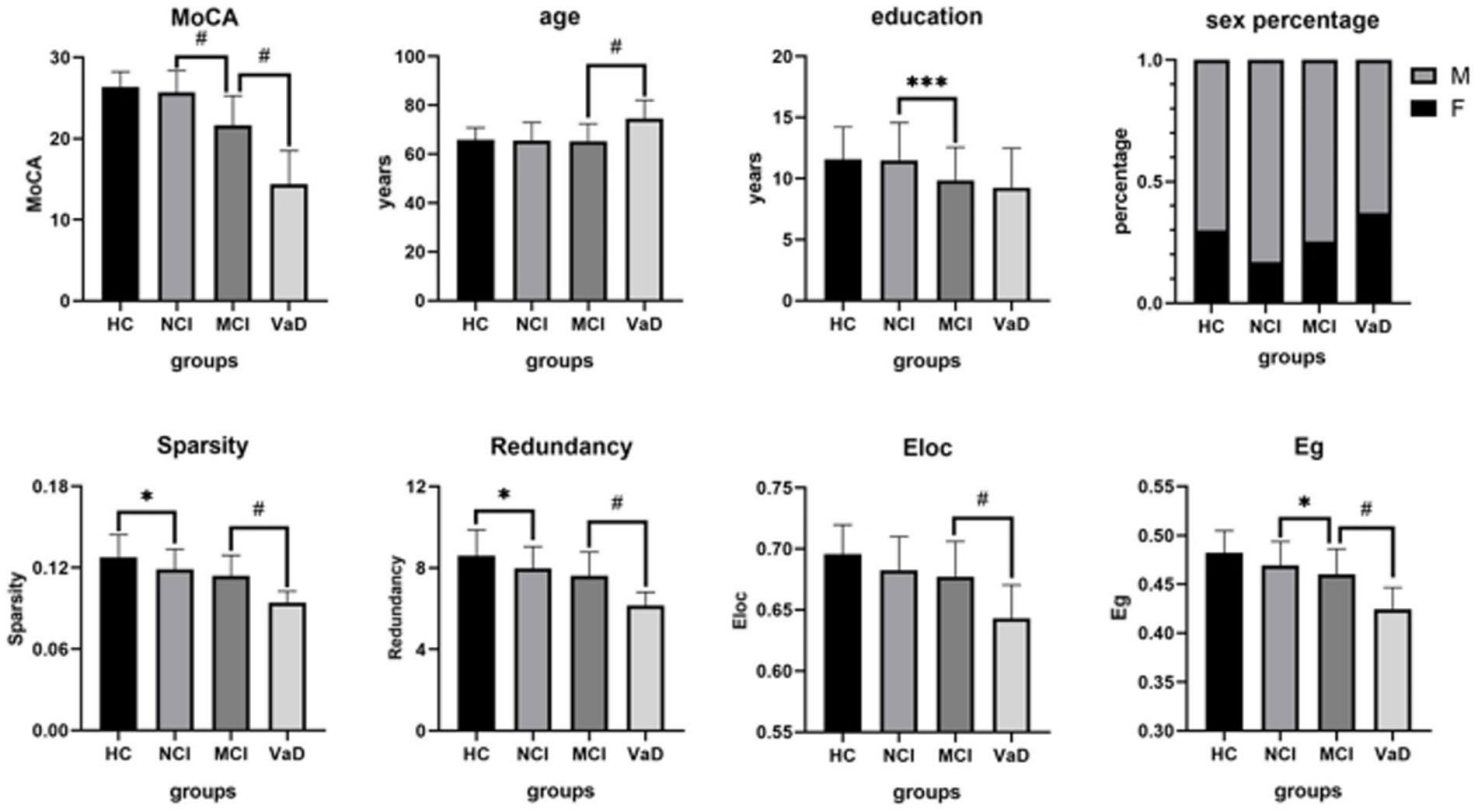
Between-group *post-hoc* comparisons of cognitive function, demographic and structural brain network characteristics. The NCI group had a lower network sparsity and redundancy than the HC group. The MCI group showed a lower MoCA, education, and network Eg than the NCI group. The VaD group demonstrated a lower MoCA and all of the network characteristics, but a higher age than the MCI group. HC, healthy control; NCI, non-cognitive impairment; MCI, mild cognitive impairment; VaD, vascular dementia; MoCA, Montreal Cognitive Assessment; Eg, global efficiency; Eloc, local efficiency; M, male; F, female.

As there were significant differences in age and education among groups (Table 1), we conducted between-group *post-hoc* comparisons between network characteristics after controlling age and education. Network sparsity and redundancy in the NCI group were significantly lower than those in the HC group (*p* = 0.0219, *p* = 0.0356, respectively), while MoCA, age, education, Eloc, and Eg showed no significant differences between these two groups (all *p*-values >0.05). MoCA, education, and network Eg in the MCI group were significantly lower than those in the NCI group (*p* < 0.0001, *p* = 0.0002, *p* = 0.0216, respectively), while age and all other network characteristics demonstrated no significant differences between the two groups (all *p*-values >0.05). MoCA and all the network characteristics in the VaD group were significantly lower than those in the MCI group (all *p*-values <0.0001). However, the age of the VaD group was significantly higher than that of the MCI group (*p* < 0.0001), while education did not differ between these two groups (*p* = 0.7531; Table 2; Figure 1).

**Table 2:** Between-group comparisons among the groups.

	HC vs. NCI	HC vs. MCI	HC vs. VaD	NCI vs. MCI	NCI vs. VaD	MCI vs. VaD
MoCA	0.7653	**<0.0001**	**<0.0001**	**<0.0001**	**<0.0001**	**<0.0001**
Age	0.9989	0.9838	**<0.0001**	0.9887	**<0.0001**	**<0.0001**
Education	0.9991	**0.0177**	**0.0143**	**0.0002**	**0.0025**	0.7531
Sparsity	**0.0219**	**<0.0001**	**<0.0001**	0.0609	**<0.0001**	**<0.0001**
Redundancy	**0.0356**	**0.0001**	**<0.0001**	0.0890	**<0.0001**	**<0.0001**
Eloc	0.0869	**0.0049**	**<0.0001**	0.4834	**<0.0001**	**<0.0001**
Eg	0.0520	**0.0001**	**<0.0001**	**0.0216**	**<0.0001**	**<0.0001**

Adjusted *p* values of pairwise post hoc comparisons. The results for network characteristics are after controlling for age and education. HC, healthy control; NCI, non-cognitive impairment; MCI, mild cognitive impairment; VaD, vascular dementia; MoCA, Montreal Cognitive Assessment; Eloc, local efficiency; Eg, global efficiency. Bold *p* values were *p* < 0.05.

### Linear regression analysis

**Figure 2:**
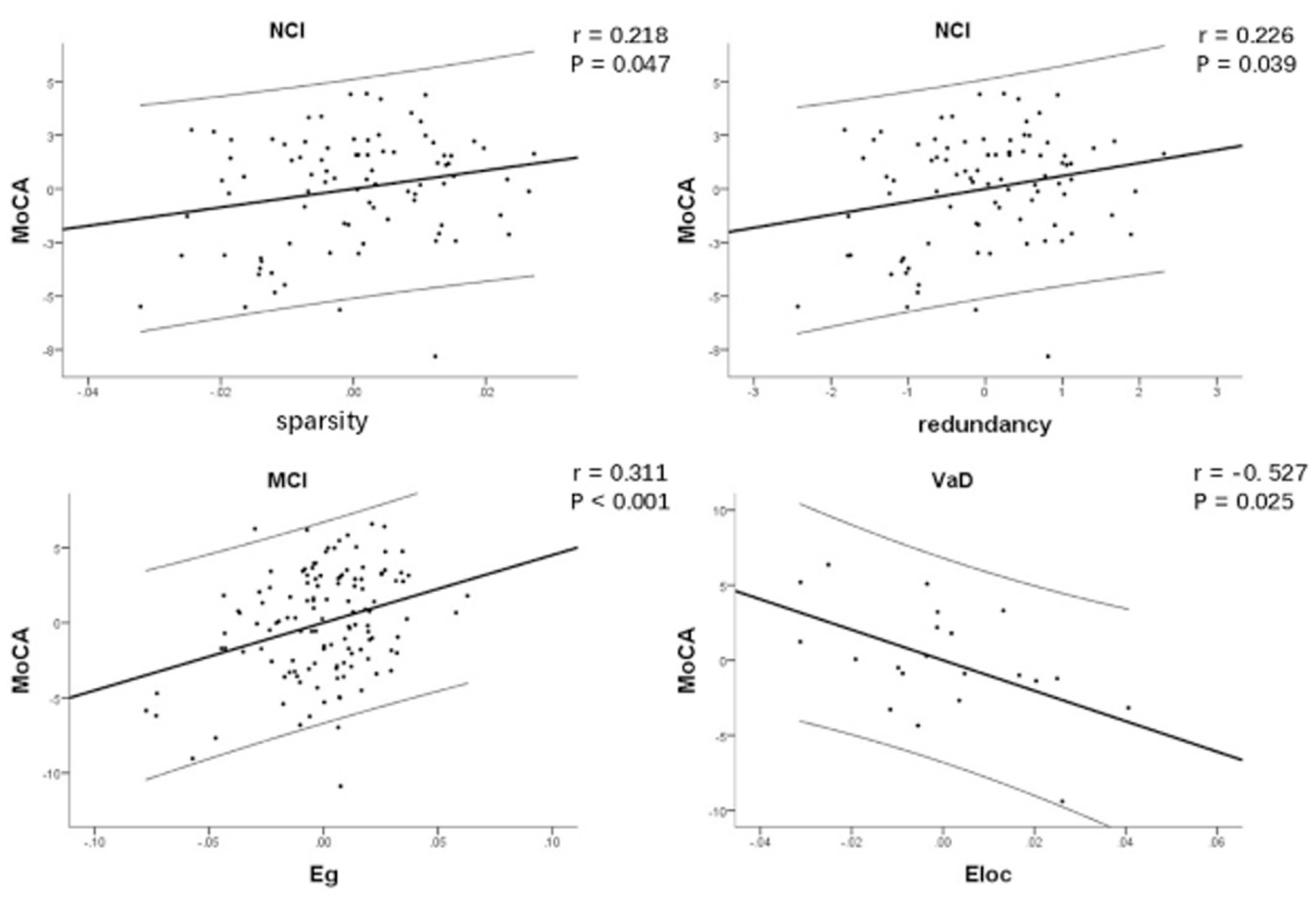
Correlation between network characteristics and MoCA after controlling for age and education within each CSVD group, respectively. There was significant positive correlation between network sparsity and MoCA within NCI group, so did redundancy and MoCA. There was significant positive correlation between Eg and MoCA within MCI group. There was significant negative correlation between Eloc and MoCA within VaD group. CSVD, cerebral small vessel disease; NCI, non-cognitive impairment; MCI, mild cognitive impairment; VaD, vascular dementia; MoCA, Montreal Cognitive Assessment; Eg, global efficiency; Eloc, local efficiency.

Network characteristics with significant group difference were considered for linear regression analysis. Table 3 and Figure 2 show the correlations between network characteristics and MoCA after controlling for age and education within each CSVD group, respectively. There was significant positive correlation between network sparsity and MoCA within NCI group (*r* = 0.218, *p* = 0.047), so did redundancy and MoCA (*r* = 0.226, *p* = 0.039). There was significant positive correlation between Eg and MoCA within MCI group (*r* = 0.311, p<0.001). There was significant negative correlation between Eloc and MoCA within VaD group (*r* = −0.527, *p* = 0.025).

**Table 3:** Linear regression analysis of MoCA within each CSVD group.

		*β*	Partial Correlation	*t*	*p*
NCI	Sparsity	0.244	0.218	2.020	**0.047**
	Redundancy	0.252	0.226	2.103	**0.039**
MCI	Eg	0.324	0.311	3.586	**<0.001**
VaD	Sparsity	−0.052	−0.057	−0.227	0.823
	Redundancy	−0.094	−0.104	−0.420	0.680
	Eg	−0.014	−0.015	−0.058	0.954
	Eloc	−0.514	−0.527	−2.483	**0.025**

Controlling for age and education. CSVD, cerebral small vessel disease; MoCA, Montreal Cognitive Assessment; NCI, non-cognitive impairment; MCI, mild cognitive impairment; VaD, vascular dementia; Eloc, local efficiency; Eg, global efficiency. Bold *p* values were *p* < 0.05.

### Correlation between network characteristics

**Figure 3:**
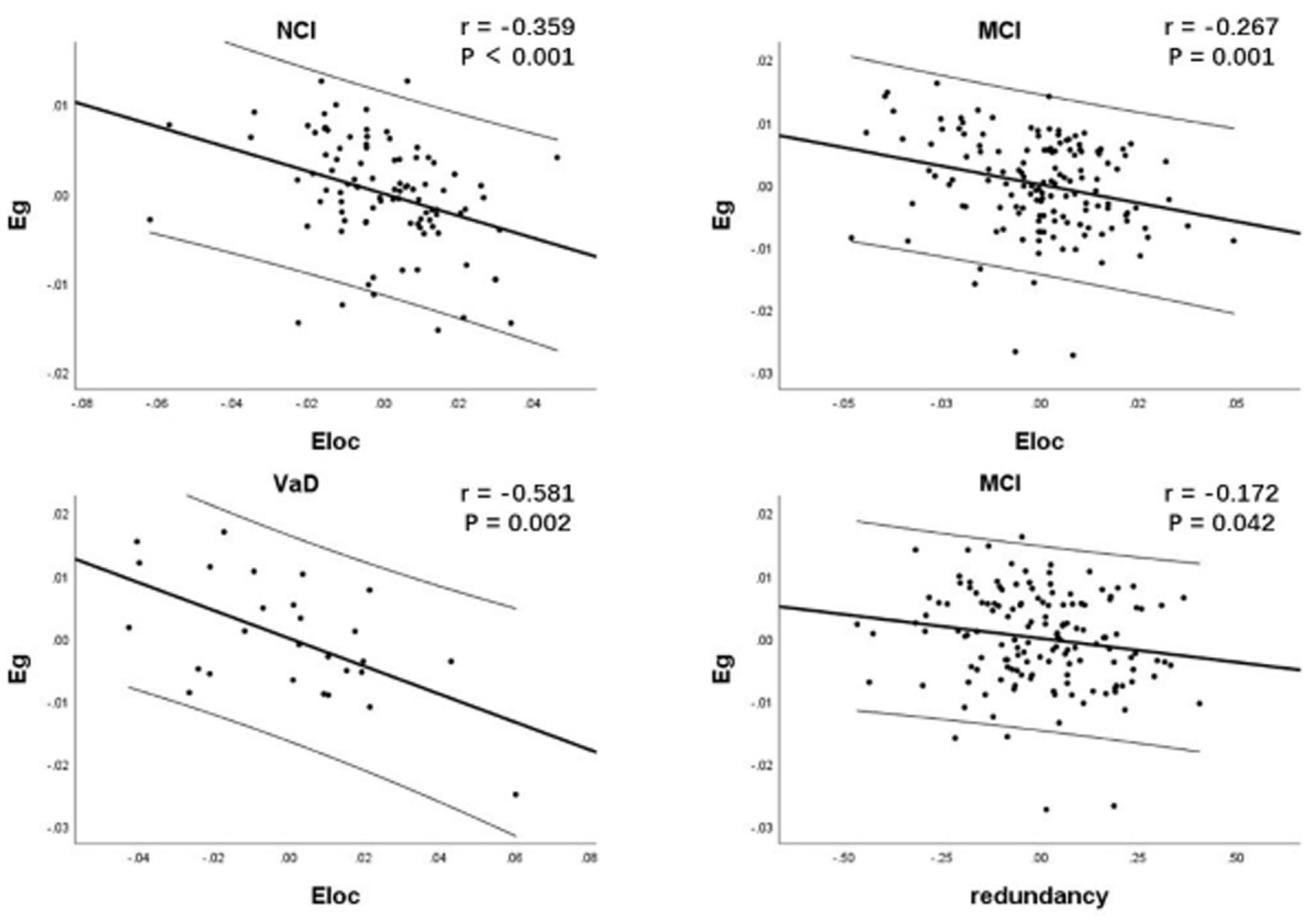
Correlation between network characteristics within each CSVD group. As network efficiency and redundancy were both implemented under the given wiring cost, partial correlation analysis was used to explore the relationship between network redundancy, Eg and Eloc by controlling for network sparsity. There was significant negative correlation between Eloc and Eg within each CSVD group. There was significant negative correlation between redundancy and Eg specifically in MCI group. CSVD, cerebral small vessel disease; NCI, non-cognitive impairment; MCI, mild cognitive impairment; VaD, vascular dementia; Eg, global efficiency; Eloc, local efficiency.

As network efficiency and redundancy were both implemented under the given wiring costs (network sparsity), partial correlation analysis was used to explore the relationship between network redundancy, Eg and Eloc by controlling for network sparsity. Due to the nonlinear changes in network characteristics at different stages of cognitive impairment, we performed partial correlation analyses for each CSVD group. Results showed that there was significant negative correlation between Eloc and Eg within each CSVD group (*r* = −0.359, *p* < 0.001; *r* = −0.267, *p* = 0.001; *r* = −0.581, *p* = 0.002, respectively). There was significant negative correlation between redundancy and Eg within only MCI group (*r* = −0.172, *p* = 0.042; Figure 3).

## Discussion

In this study, we explored the hypothesis that alterations in brain structural network across different stages of cognitive impairment in CSVD display heterogeneity, indicative of the robustness of a balanced network topology.

Intergroup analysis revealed significant differences in network characteristics among HC and different CSVD groups. The NCI group exhibited a significant reduction in sparsity and redundancy compared to the HC group, indicating that CSVD-related damage primarily affected redundant connections during the early stages of the disease. Eloc and Eg showed no statistically significant changes in the NCI group compared to the HC group, implying that network integration and segregation remained relatively unaltered during the early stages of CSVD. Consequently, the cognitive function appeared to be maintained within normal parameters. The alterations observed in the brain structural network topology within the NCI group align with the principles of the brain reserve theory. Brain reserve is typically conceptualized as neurobiological capital that enables individuals to effectively withstand brain damage before clinical or cognitive manifestations become evident (Stern et al., 2020). Network redundancy appeared to function as a brain reserve factor in the early stage of CSVD, exhibiting resilience against cognitive impairment. The reduction in redundant connections could arise from either compensatory mechanism responding to impaired effective connections or potentially result from random attacks inflicted upon the brain structural network by CSVD-related brain injury. In either scenario, it is theoretically conceivable that redundant connectivity may offer a degree of delay in the onset of cognitive impairment associated with CSVD-related brain damage. Further investigations, such as longitudinal studies, are needed for substantiating this hypothesis. In the MCI group, Eg exhibited a significant decrease when compared to the NCI group, indicating a disruption in network integration that potentially contributes to cognitive impairment. The association between the decline of brain structural network Eg and CSVD related cognitive impairment has been consistently reported in previous studies (Boot et al., 2020; Lawrence et al., 2014; Tuladhar et al., 2016b). For example, a cross-sectional study found that both Eg and Eloc were significantly disrupted in CSVD and associated with cognitive function. The association of Eg with cognition were stronger than other MRI measures including conventional DTI measures (Lawrence et al., 2014). Longitudinal study showed that patients developed dementia had lower Eg and Eloc at baseline as compared with the group without dementia. Lower Eg increased the risk of incident all-cause dementia (Tuladhar et al., 2016b). In alignment with prior research, our study highlights a significant reduction in Eg within the MCI group when compared to the NCI group. However, marginal changes were observed in redundancy and Eloc. These findings might suggest the preservation of reserve connections and localized information exchange within the brain network in MCI group. These factors may contribute to the relative preservation of cognitive function in individuals with MCI, potentially delaying the onset of dementia. The observation that Eg and Eloc exhibit distinct impacts on cognitive change diverges from the outcomes of prior studies, which considered Eg and Eloc as homogenous network characteristics (Boot et al., 2020; Lawrence et al., 2014; Tuladhar et al., 2016a). Drawing from topological theory, Eloc is indicative of the network’s resilience against random attacks, suggesting its potential protective role in cognitive function. Prior studies did not thoroughly consider the interplay among different network attributes within the context of wiring costs. Further exploration of the relationships among these attributes can provide insights into the hypothesis regarding the protective influence of Eloc and redundancy. In our present study, we regulated network sparsity in consideration of wiring costs. The results showed that individuals with lower Eg exhibited relatively higher Eloc within each CSVD group and higher redundancy within MCI group, which substantiated our supposition. These observations signify the presence of brain reserve or compensation mechanisms, characterized by alternative connections and enhanced local information exchange to mitigate potential disruptions. This hypothesis needs to be confirmed by more compelling and direct evidence, such as by observing the dynamic changes in these network characteristics by conducting longitudinal studies in the future. All of the network characteristics were decreased in the VaD group compared to the MCI group, which indicated severe destruction of the network. This result posited that a reduction in Eloc and redundancy could also potentially play a pivotal influence on the pathogenesis of dementia in CSVD, thus extending the findings of prior research that emphasized the sustained decrease in Eg as a significant contributing factor to dementia development in CSVD (Tuladhar et al., 2016b). The collective reduction in sparsity, redundancy, Eg, and Eloc could suggest the depletion of brain reserve, potentially signifying the irreversibility of VaD.

We found significant correlations between network characteristics and cognitive function at different stages of CSVD. In the NCI group, network sparsity and redundancy exhibited a significant positive correlation with MoCA. This suggests that, even in the early stages of CSVD when patients have not yet exhibited significant cognitive impairment, the overall cognitive performance of individuals remains contingent on the structural organization of the brain network. The findings in the NCI group are of particular significance, as prior studies predominantly concentrated on CSVD cases characterized by pronounced clinical cognitive impairment. The concept of a preclinical stage of cognitive impairment has been introduced to describe CSVD patients without evident cognitive impairment symptoms despite the substantial burden of pathological lesions, who remain at a high risk of transitioning to MCI (Wardlaw et al., 2013a). The findings in the NCI group within this study offer valuable insights into this concept. No significant correlations were observed between Eg and MoCA, as well as between Eloc and MoCA within NCI group. This could be attributed to the initial loss of redundant connections in the early stages of CSVD, which, in turn, delays the reduction in network efficiency. It is not until the disease progresses to the MCI stage that the decline in Eg starts to make a noteworthy contribution to cognitive impairment. In the MCI stage, the influence of brain network sparsity and redundancy on cognitive function diminishes, while network redundancy exhibits a degree of counteraction to the decline of Eg. The phenomenon of changes in brain network redundancy not being synchronized with the decline in Eg in both NCI and MCI groups may be attributed to the brain’s capacity to adapt its internal network structure in response to varying degrees of injury. However, during the VaD stage, there was no discernible linear correlation between the sparsity, redundancy, and Eg of the brain network and cognitive function. This observation suggests that the structure of the brain network becomes increasingly disorganized in VaD stage. Notably, Eloc displayed a significant negative correlation with cognitive function, signifying that worse cognitive function was associated with higher local efficiency. This observation may suggest that, with the exacerbation of cognitive impairment in the VaD stage, the brain network structure tends to become increasingly segregated. Theoretically, the capability of segregated information processing not only allows for flexible and rapid reconfiguration of the brain network in response to various task demands, but also benefits network robustness (Sporns and Betzel, 2016; Wig, 2017). The seemingly paradoxical negative correlation between Eloc and cognitive function in this study can be elucidated within the framework of the trade-off theory, which is postulated as a fundamental principle of the human connectome. This theory suggests that there are trade-offs among wiring costs (represented by network sparsity), integration (represented by Eg), and segregation (represented by Eloc) within the human brain network (Bullmore and Sporns, 2012; Ma et al., 2023). Effective communication within the human brain necessitates not only efficient global information integration but also the ability for segregated information processing, which is a key element of the small-worldness concept (Sporns, 2013). For a given wiring cost, a competition may arise between Eloc and Eg. Hence, in the context of a substantial reduction in wiring costs, the significant negative correlation between Eloc and cognitive function observed within the VaD group suggested that the brain is counterbalancing Eg by enhancing Eloc. The more pronounced this trade-off, the more severe the cognitive impairment. Through partial correlation analysis (while controlling for wiring cost), we detected a trade-off relationship between Eg and Eloc at different stages of cognitive impairment in CSVD. This finding indicates that the disequilibrium between brain network integration and segregation may persist throughout the entire course of the disease.

Our findings, as derived from intergroup comparisons and correlation analyses, corroborate our initial hypothesis regarding the heterogeneous alterations in the brain structural network across different stages of cognitive impairment in CSVD. By meticulously examining the wiring cost, this investigation delves into the intricate relationships among the attributes of the brain structural network. These discoveries provide empirical support for the theories related to brain reserve and the concept of a brain network trade-off. The robust network system in CSVD reaffirms the notion that the analysis of brain structural networks is a promising method for evaluating cognitive impairment caused by CSVD, surpassing traditional macroscopic imaging assessments. Although previous studies have demonstrated the advantages of structural network efficiency in assessing cognitive impairment due to CSVD, and the complete mediating effect of network efficiency between traditional MRI features and cognitive impairment, they have overlooked the roles of reserve and compensation in neuroprotection, as well as the complex relationships among network characteristics (Boot et al., 2020; Du et al., 2019; Lawrence et al., 2014). Our present study filled this gap in CSVD research.

This study presents several limitations. First and foremost, it is essential to acknowledge that this research was cross-sectional in nature and featured relatively modest sample sizes in certain CSVD groups. Consequently, the conclusions reached herein warrant further validation through longitudinal studies involving more extensive participant cohorts. Second, it is worth noting that the absence of a universally agreed-upon methodology for defining nodes and connection weights when constructing brain structural networks based on DTI introduces the potential for methodological bias, as distinct approaches to network construction can yield varying research outcomes. Third, despite employing comprehensive clinical histories, imaging analyses, and neuropsychological assessments to mitigate the influence of AD, it is impossible to entirely rule out the impact of mixed dementia in this study. Lastly, given the diverse presentations of cognitive dysfunction arising from CSVD, there is a need for subsequent studies to concentrate on specific cognitive domains through subnetwork analysis. This necessitates the inclusion of larger sample sizes encompassing CSVD-related cognitive impairment across different cognitive domains.

## Conclusion

In summary, this study furnishes evidence of heterogeneous alterations within the brain structural network across distinct stages of cognitive impairment in CSVD. At the NCI stage, the disconnectivity of the brain structural network predominantly manifests as the loss of redundant connections. In contrast, at the MCI stage, the decline in Eg takes on paramount significance in its contribution to cognitive impairment, while network redundancy, regarded as a form of brain reserve, presents a certain degree of counteraction to the decline in Eg. The prominent manifestation in the VaD stage encompasses a decline in all network characteristics. Notably, there are trade-offs among brain network wiring cost, integration, and segregation across the various stages of cognitive impairment in CSVD.

## Data Availability

The raw data supporting the conclusions of this article will be made available by the authors, without undue reservation.

## Footnotes

^1^ https://www.nitrc.org/projects/panda
